# Using a Bone-Conduction Headset to Improve Speech Discrimination in Children With Otitis Media With Effusion

**DOI:** 10.1177/2331216519858303

**Published:** 2019-08-29

**Authors:** Tamsin Holland Brown, Marina Salorio-Corbetto, Roger Gray, Alexandra James Best, Josephine E. Marriage

**Affiliations:** 1 Cambridge Community Health Trust, UK; 2 Children’s Hearing Evaluation and Amplification Resource, Shepreth, Royston, Hertfordshire, UK; 3 Auditory Perception Group, Department of Psychology, University of Cambridge, UK; 4 Cambridge University Hospitals NHS Foundation Trust, Cambridge, UK

**Keywords:** assistive listening devices, bone conduction, glue ear, otitis media with effusion, watchful waiting

## Abstract

The recommended management for children with otitis media with effusion (OME) is ‘watchful waiting’ before considering grommet surgery. During this time speech and language, listening skills, quality of life, social skills, and outcomes of education can be jeopardized. Air-conduction (AC) hearing aids are problematic due to fluctuating AC hearing loss. Bone-conduction (BC) hearing is stable, but BC hearing aids can be uncomfortable. Both types of hearing aids are costly. Given the high prevalence of OME and the transitory nature of the accompanying hearing loss, cost-effective solutions are needed. The leisure industry has developed relatively inexpensive, comfortable, high-quality BC headsets for transmission of speech or music. This study assessed whether these headsets, paired with a remote microphone, improve speech discrimination for children with OME. Nineteen children aged 3 to 6 years receiving recommended management in the United Kingdom for children with OME participated. Word-discrimination thresholds were measured in a sound-treated room in quiet and with 65 dB(A) speech-shaped noise, with and without a headset. The median threshold in quiet (*N* = 17) was 39 dB(A) (range: 23–59) without a headset and 23 dB(A) (range: 9–35) with a headset (*Z* = −3.519, *p* < .001). The median threshold in noise (*N* = 19) was 59 dB(A) (range: 50–63) without a headset and 45 dB(A) (range: 32–50) with a headset (Z = −3.825, *p* < .001). Thus, the use of a BC headset paired with a remote microphone significantly improved speech discrimination in quiet and in noise for children with OME.

## Background

Otitis media with effusion (OME), also called ‘glue ear,’ is caused by viral upper respiratory tract infections, ear infections such as acute otitis media, and chronic Eustachian tube dysfunction. OME is common in early childhood. The proportion of children affected is greater in winter compared with summer and decreases with age. For example, from February to August, prevalence changes from 37% to 16% in 8-month-old children and from 16% to 3% in 61-month-old children affected (Midgley, Dewey, Pryce, Maw, & ALSPAC Study Team, 2001). This reflects the fluctuating nature of OME. OME is more frequent in some population groups such as children with Down’s syndrome (Austeng et al., 2013) and children with cleft palate (Flynn, Möller, Jönsson, & Lohmander, 2009; Paradise, Bluestone, & Felder, 1969).

Despite its transitory nature, OME can have long-lasting consequences. The presence of fluid in the middle ear causes conductive deafness, with approximately 70% of children with chronic OME developing mild-to-moderate hearing loss (HL) that typically fluctuates (Daly, Hunter, & Giebink, 1999). This level of HL impairs the discrimination of speech in both quiet and in noise (Cai & McPherson, 2017). Chronic fluctuating HL increases the risk of educational difficulties (phonics, reading, and spelling) (Bennett, Haggard, Silva, & Stewart, 2001; Catts, Fey, Tomblin, & Zhang, 2002; Scarborough & Dobrich, 1990; Silva, Chalmers, & Stewart, 1986; Silva, Williams, & McGee, 1987) and adversely affects speech (Silva et al., 1986) and language development (Holm & Kunze, 1969; Silva et al., 1986). In addition, OME is associated with auditory processing deficits (Jerger & Musiek, 2000; Polley, Thompson, & Guo, 2013), self-esteem and behavioral problems (Bennett & Haggard, 1999; Silva et al., 1986), hyperactivity (Hagerman & Falkenstein, 1987), attention-deficit hyperactivity disorder (Adesman, Altshuler, Lipkin, & Walco, 1990; Padolsky, 2008), learning disability (Padolsky, 2008), and sociocommunicative difficulties (Tajima-Pozo et al., 2010). The inconsistent auditory input due to the fluctuating HL associated with OME can lead to changes in the typical development of speech cue weights related to formant transitions (Nittrouer, 1996a; Eapen et al., 2008). Weighting strategies are thought to be used to assess the phonemic structure, which in turn is used to store and retrieve information in verbal working memory, and this is important for sentence comprehension (Nittrouer & Burton, 2005). In fact, children with a history of OME can have poorer phonemic awareness (Nittrouer, 1996b; Nittrouer & Burton, 2005), sentence comprehension (Nittrouer & Burton, 2005), and working memory (Nittrouer & Burton, 2005) than their peers with no history of OME from non low socioeconomic backgrounds. Children at preschool (age 3–4) or reception class (age 4–5) or Year 1 (age 5–6) at school will be concentrating on phonics and starting to learn to read and learning specific language skills at a critical time in education. At this time, children cannot read to mitigate the impact of hearing impairment on their learning. The developmental sequelae of early middle-ear disease, especially deficits in reading abilities, if present, may remain significant even in late childhood and early teens, as shown by Bennett et al. (2001).

In the United Kingdom, children with OME are monitored with watchful waiting and advice until it resolves, or grommets are considered for those with “persistent bilateral OME documented during a period of 3 months with a hearing level in the better ear of 25 to 30 dBHL or worse averaged across 0.5, 1, 2, and 4 kHz (or equivalent dB(A) when HL is not available)” (National Collaborating Centre for Women’s and Children’s Health, 2008). There are often months between hearing tests and referrals to specialists. During this time, mild-to-moderate HL often persists and significantly affects the everyday life of children and their families; 60% of parents of children with mild-to-moderate HL report that they need more support for their child (Archbold et al., 2015).

One way of supporting children with OME could be by providing amplification via hearing aids. Air-conduction (AC) hearing aids provide amplification based on AC hearing thresholds. However, because AC thresholds fluctuate over time (Khanna, Lakhanpaul, & Bull, 2008; Midgley et al., 2001), frequent follow-up is needed in order to avoid overamplification (i.e., often an unfeasible number of appointments per child). In addition, ear molds for AC hearing aids need to be frequently renewed due to growth. Alternatively, bone-conduction (BC) devices do not require a frequent follow-up because BC hearing thresholds do not vary significantly over the course of OME, and they do not require ear molds. However, while these devices are effective to treat conductive HL in children (McDermott, Williams, Kuo, Reid, & Proops, 2009), anecdotal evidence suggests that they can cause discomfort due to the pressure of the transducer against the skin and the elastic band often used as support. In addition, the cost of both AC hearing aids and BC hearing aids is high. Given the high prevalence of OME and the transitory nature of the HL associated with it, cost-effective solutions are needed.

In recent years, the leisure industry has developed affordable BC headsets that are worn around the back of the head and hook over the top of the ears with the BC transducer resting on the zygomatic arch (cheekbones) bilaterally. Vibration passes through the bone to the cochleae by-passing the fluid-filled middle ear. These headsets are normally sold as sport headphones so that runners or cyclists can hear music or telephone calls through the headphones connected via Bluetooth to their phone but also to allow their ear canals to remain uncovered to listen to traffic noises for safety. Such a removable, small, one-size-fits-all bone-conducting headset coupled with a Bluetooth microphone has the potential to become an affordable solution to aid children with OME during the class or in other one-to-one situations.

The objective of this study was to assess the effectiveness of wearing a BC headset paired with a Bluetooth microphone on the discrimination of distant speech in quiet and in noise.

## Methods

### Participants

Nineteen children aged 3 to 6 years (median age 52 months, range: 38–80) participated. Children were recruited at their first Community Pediatric Audiology appointment between 2016 and 2018 to take part in a study exploring interventions over the watchful waiting period for children with HL secondary to chronic OME (*B*one conduction *I*n *G*lue ear study; ISRCTN13818722). Inclusion criteria at recruitment were bilateral OME, with an AC HL worse than 25 dBHL across three frequencies in the better hearing ear, ‘normal’ BC with thresholds better than 20 dBHL and type-B tympanograms (Jerger, 1970), indicating little change in compliance for a wide range of pressure variation. Exclusion criteria were the presence of sensorineural HL, a diagnosed medical syndrome (such as developmental delay, autism spectrum, or cleft palate), or a main language that was not English. National Health Service Ethical approval was obtained from West Midlands Research Ethics Service (Integrated Research Application System [IRAS] Project ID: 190096, REC Reference 15/WM/0438). Informed consent was obtained from parents and children. Families had their travel expenses reimbursed. Children were given a toy and a certificate to acknowledge their participation.

### Basic Hearing Evaluation

An otoscopic examination was carried out using a Welch Allyn 3.5v Otoscope. Tympanometry was performed with a Grason-Stadler GSI 39 middle-ear analyzer using a 226 Hz probe. Conditioned-play audiograms were obtained using a Kamplex KC35 or a Tes1350A audiometer and TDH 39-P headphones for AC and a Radioear B-71 transducer for BC. Thresholds for both AC and BC were measured at 500, 1000, 2000, and 4000 Hz. In cases where headphones were not accepted by the child, sound-field AC thresholds were obtained using a Kamplex AC5 pediatric warble tone generator.

### BC Headset and Bluetooth Microphone

**Figure 1.**
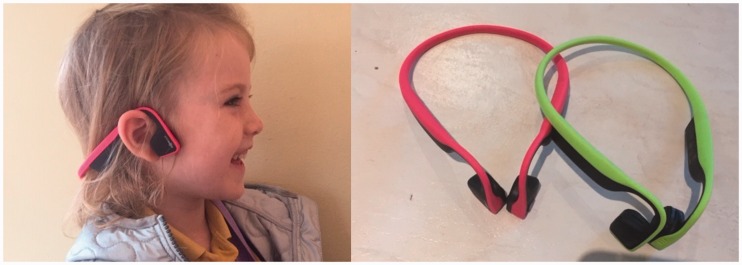
Left: Bone-conduction headset placed on a user. Photograph published to illustrate the use of the headset (i.e., it is not a photograph of a study participant). Consent for publication was obtained from the parents. Right: bone-conduction headsets.

The Aftershokz BC headset (Figure 1) was used, which is a lightweight device (41 g) composed of two BC transducers joined by an arch that goes around the back of the neck. Each of the transducers rests on each zygomatic arches of the users without exerting pressure. The headset has a built-in battery that is rechargeable within 2 hr and a continuous play time of 6 hr. Aftershokz uses Bluetooth to receive signals from external sources and has a wireless range of 10 m. A Sena Bluetooth microphone was paired to the headset in order to convey the speech signal to the children. The Sena high-fidelity lapel microphone is commonly used for intercommunication when riding motorbikes. It has a sampling rate of 48000 Hz and uses a proprietary noise-reduction algorithm. It weighs 30 g, has a rechargeable battery, and a talk time of 6 hr. It has a wireless range of 350 m. Listening checks were performed each time the headset was used to verify that the speech signal was being transmitted to the headset. The volume of the headset was set to four steps below the maximum level and each child was asked whether they thought that the sound level was comfortable, which they did.

### Automated McCormick Toy Test

An automated version of the McCormick Toy Test (MCTT; McCormick, 1977) was presented in quiet and in background noise in order to determine the speech level that led to 71% correct speech discrimination, termed ‘word-discrimination threshold,’ (WDT) as used by Summerfield, Palmer, Foster, Marshall, and Twomey (1994) and implemented by the manufacturer in the equipment used here, a Phoenix Automated Speaker system (Soundbyte Solutions Ltd.). The test was performed inside an acoustically treated room. Children sat in front of a small table where the toys corresponding to the test items were laid out. The test items were as follows: *cup*, *duck*, *spoon*, *shoe*, *man*, *lamb*, *plate*, *plane*, *horse*, *fork*, *key*, *tree*, *house*, and *cow*. It should be noted that each item has a matching word that has a similar vowel or diphthong, but different consonants (McCormick, 1977), for example *plate-plane*. After checking that the child was familiar with the vocabulary used in the test, they were instructed to point at the toy requested in each trial. An experienced audiologist operated the Phoenix Automated Speaker system. A practice run was performed by the audiologist using her own voice in order to verify that the children could do the test. Next, prerecorded stimuli and carrier phrases were used where the talker was a female speaker. On each trial, an item was randomly selected and presented after a carrier phrase, for example, ‘Where’s the lamb?’ The WTD was adaptively estimated in two stages: (a) A homing stage that determined the appropriate level range for testing using two reversals following a one-up one-down procedure with a step size of 12 dB and (b) A testing stage that determined the final outcome using a two-down one-up procedure with 6-dB steps and six reversals. Testing with the Phoenix can be stopped by the operator at any time. The outcome of the test is updated with each turn point. In cases where it was predicted that the useful time to test the participant was short (due to a short attention span, etc.), five (in one case) or three turn points (in four cases) were obtained. This was kept consistent across conditions at the individual level. The loudspeaker of the Phoenix was placed 3 m away from the child, at an azimuth of 0° and roughly at the height of the child’s head. The distance between the loudspeaker and the child was similar to that between teacher and pupil when the pupil is in the first row of the class, as is recommended during watchful waiting. A Tecpel 330 type II sound level meter was used to calibrate the speech level to be 60 dB(A) at 1 m from the loudspeaker, following the calibration method recommended by the manufacturer. However, children were sitting at 3 m from the loudspeaker. Thus, levels were checked at the position of the child’s head to estimate the actual presentation level. The level of speech measured at the position of the child’s head was 9 dB below the sound level measured at 1 m from the loudspeaker. In what follows, word-discrimination thresholds are expressed as sound level measured at the position of the children’s head. Four conditions were tested, resulting from two headset conditions (with or without headset) and two backgrounds (quiet and speech-shaped noise). For the tests with the headset on, a Sena microphone that had been paired to the Aftershokz headset was placed in front and slightly below the loudspeaker, simulating a location on the lapel of a teacher. Speech-shaped noise was generated using a Kamplex KC35 audiometer and delivered via a JVC loudspeaker placed at 2.1 m from the child, with an azimuth of 45°. The level of the noise was calibrated to be 65 dB(A) at the position of the child’s head and was kept constant during the test, except for one child for whom the noise level was reduced by 5 dB for comfort. The noise level was chosen to be representative of the noise level in a standard classroom in use (Jamieson, Kranjc, Yu, & Hodgetts, 2004).

For each child, all four conditions of the MCTT first in quiet and then in noise, and first without the headset and then with the headset, were tested in the same session. Conditions were ordered in increasing complexity due to the young age of the participants. Only one run was obtained in each condition. This meant that children would have a practice run plus four runs of the test. It was necessary to limit the number of runs to one per condition to ensure a complete data set from as many children as possible given their young age and, in some cases, their short attention span.

## Results

### Basic Hearing Evaluation

Otoscopic examination revealed bilateral OME for all children except in one case of unilateral microtia and atresia. OME was unilateral in this case. OME was complicated with tympanic perforation in two cases, one of which was bilateral.

**Figure 2.**
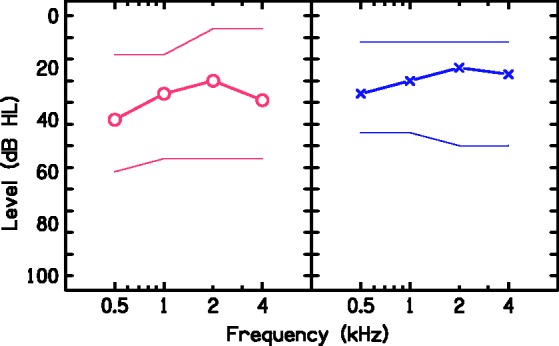
Median hearing thresholds for each test frequency for the right (open circles) and the left (crosses) ears. The top line joins the minimum hearing thresholds for each test frequency and the bottom line joins the maximum hearing thresholds for each frequency.

Hearing thresholds were measured on the day the MCTT was performed for nine children. However, this was not possible for the other 10 children. Overall, the median time difference between the audiogram and the MCTT was 1.2 weeks (range: 0–23.7). Ear-specific thresholds for octave frequencies between 500 to 4000 Hz were available for all but one child. Excluding this child, the median four-frequency average threshold was 32 dBHL (range: 11–50) for the right ears and 29 dBHL (range: 11–40) for the left ears. Most children had similar average hearing thresholds across ears, but there were six children who had differences greater than 10 dB across ears. In these cases, interaural differences in the average thresholds were 14, 15 (two cases), 16, 17, and 28 dB. For the one child where ear-specific thresholds were not available, sound-field thresholds were available. For comparison, sound-field thresholds measured in dB(A) were converted to dBHL following the procedure recommended by the British Society of Audiology (2008). Converted thresholds for 500, 1000, 2000, and 4000 Hz were 40, 30, 40, and 40 dBHL, respectively. Figure 2 shows the median ear-specific audiometric AC thresholds for each test frequency for the group. The range of the hearing thresholds is also shown. Although children had audiometric AC thresholds ≥25 dBHL or worse for at least three of the test frequencies at the time of recruitment, there were some children who did not meet this criterion at the time of testing or according to their most recent audiogram at the time of testing. Six of the children had normal hearing thresholds at least in one ear on the day when the MCTT was tested or according to their most recent audiogram. Similarly, although most children had type-B tympanograms (including the three cases of tympanic perforation for whom the ear canal volume was large, as expected), five of the children who had normal hearing thresholds around the time of testing had type-C tympanograms at least unilaterally. This is due to the fluctuating nature of OME.

### Word-Discrimination Thresholds

**Figure 3.**
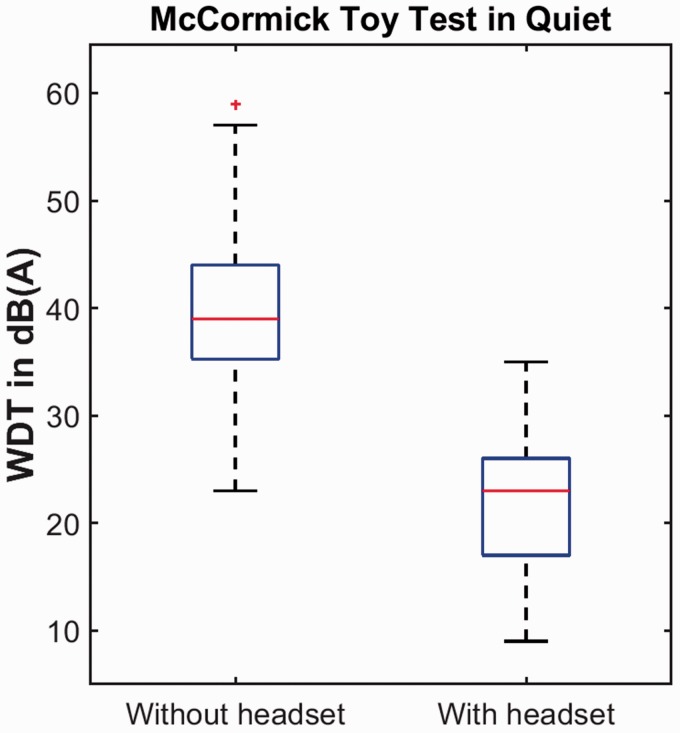
Word-discrimination thresholds (WDTs) in quiet measured with a headset and without a headset. The boxes represent interquartile ranges and medians are indicated by red lines. Red crosses represent outliers. Whiskers represent the 25^th^ and 75^th^ percentiles minus and plus up to 1.5 times the interquartile range, respectively.

**Figure 4.**
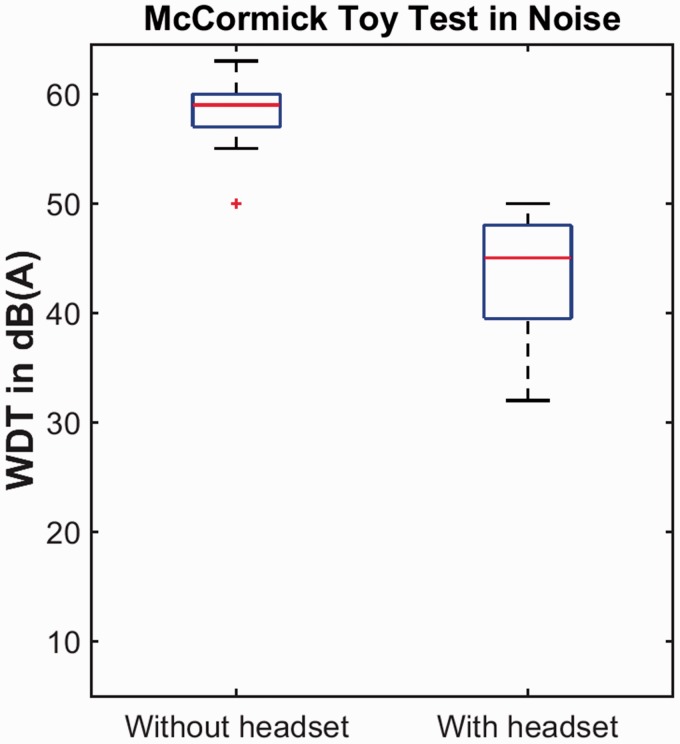
As Figure 3 but for word-discrimination thresholds in noise.

Due to an error, two children did not complete the test in quiet. Thus, results are available for 17 children in quiet and for 19 children in noise. In one case, for the test in noise, the outcome could not be determined without the headset as the percentage of discrimination was lower than 71% at the maximum presentation level produced by the Phoenix: 72 dB(A) at 1 m from the speaker, that is, 63 dB(A) at the position of the head of the child.^1^ We assigned this highest possible value to this data point for the analysis, although the true outcome would have been greater than this. Figure 3 shows the WDTs in quiet, and Figure 4 shows the WDTs in noise. The effect of the headset was assessed separately in quiet and in noise. Because data were not normally distributed, paired samples were compared using a two-tailed Wilcoxon Matched-Samples Signed Rank test in each case. For the MCTT in quiet, the median threshold was 39 dB(A) (range: 23–59) without a headset and 23 dB(A) (range: 9–35) with a headset, and the difference between these two thresholds was statistically significant (*Z* = −3.519, *p* < .001). For the MCTT in noise, the median threshold was 59 dB(A) (range: 50–63) without a headset and 45 dB(A) (range: 32–50) with a headset, and the difference was statistically significant (*Z* = −3.825, *p* < .001). In all individual cases, except for one case, word-discrimination thresholds were better with a headset than without a headset. In one case, there was no difference in the WDT with and without a headset in quiet. The median improvement of the word-discrimination thresholds with a headset was 18 dB in quiet and 13 dB in noise.

Outcomes were reanalyzed using only data obtained from children whose hearing thresholds were raised at the time of testing. Recall that six children had normal hearing thresholds at the time of testing or according to their most recent audiograms. When these children were excluded the median word-discrimination threshold in quiet was 41 dB(A) (range: 32–59) without a headset and 24 dB(A) (range: 17–35) with a headset, and the difference was still statistically significant (*Z* = −2.940, *p* = .003). The median word-discrimination threshold in noise was 60 dB(A) (range: 55–63) without a headset and 45 dB(A) (range: 32–50) with a headset, and the difference was still statistically significant (*Z* = −3.181, *p* = .001).

## Discussion

### Effect of the Headset on the Word-Discrimination Thresholds in Quiet and in Noise

We have assessed the effect of wearing a BC headset paired with a Bluetooth microphone on the discrimination of distant speech in quiet and in noise for children with OME. The experimental setup aimed to be representative of a noisy classroom where the student was sitting 3 m away from the teacher and the noise was spatially separated, as it would be when generated by the other children.

The median four-frequency average threshold was 32 dBHL (range: 11–50) for the right ears and 29 dBHL (range: 11–40) for the left ears. Sabo, Paradise, Kurs-Lasky, and Smith (2003) tested a large group of children including children of similar age to our sample (27–65 months) who had OME. The average four-frequency thresholds reported for children with bilateral OME were 26.2 dBHL for the right ears and 25.7 dBHL for the left ears. For comparison, the mean of the thresholds obtained here was calculated and found to be 32 dBHL for the right ears and 26 dBHL for the left ears. The mean thresholds for the right ears are slightly higher in this study. Excluding the two children with tympanic perforation and the child who had unilateral microtia and atresia, which could have raised the mean for the group, the mean four-frequency average thresholds were 30 and 25 dBHL for the right and left ears, respectively.

The median word-discrimination threshold in quiet without a headset was 39 dB (A) (range: 25–59). Only three children achieved a word-discrimination threshold of 35 dB(A) or lower, which corresponds to an average threshold of 20 dBHL and it is considered normal as determined by Summerfield et al. (1994), who tested a large group of children in a clinical environment similar to the one used here. With a headset, the median word-discrimination threshold was reduced to 23 dB(A) for the group. Individual word-discrimination thresholds fell in the range between 9 and 35 dB(A). WDTs were significantly better with a headset than without a headset.

This study raises the possibility of supporting children throughout periods of fluctuating HL due to OME over the watchful waiting period with a simple, affordable, easily available, off-the-shelf, BC headset paired via Bluetooth to a microphone. These headsets could be used to help children to hear quiet speech sounds particularly in situations where there is background noise or in other difficult listening environments. Children could wear the headset as needed, for example, for speech and language therapy sessions or phonics lessons at school or chatting when the child is in the back of a car or on a bicycle ride. The headsets seemed popular with all the children in the study. In summary, the headset could be used in the context of short-term care while children are under follow-up in order to decide whether grommets or a long-term audiological care plan is needed. In these conditions, the headset could be used to support children with OME at a critical time in their development, potentially reducing the occurrence of developmental problems arising as a result of HL. This is yet to be determined.

### Limitations

A possible issue when obtaining a single run in each condition is that differences across conditions for each child are due to random variability. For the MCTT in quiet, a WDT difference of 7 dB across conditions is considered clinically relevant, with differences occurring due to chance only once in 20 times (Summerfield et al., 1994). There were only two children from our sample for whom differences were 0 and 6 dB, respectively. All other participants had differences that ranged between 9 and 42 dB. For the MCTT in noise, differences smaller than 6.8 dB could occur when testing children with a single run of the test just due to chance variation (Lovett, Summerfield, & Vickers, 2013). Again, only one of our participants had a difference across conditions with and without a headset that was smaller than 6.8 dB, at 6 dB. For all other participants, differences across conditions with and without a headset ranged between 8 and 27 dB. This suggests that it is unlikely that, overall, differences across conditions with and without a headset, both in quiet and in noise, were the result of random variation. Reliability, both in quiet and in noise, decreases with decreasing number of reversals up to a value where scores stabilize. For most participants, we conducted the MCTT using six reversals, but given the short attention span of some participants, we used five or three reversals in some cases. Reliability is quantified as the within-subjects standard deviation, with larger standard deviations corresponding to lower reliability. According to Summerfield et al. (1994), the increase in the within-subject standard deviation when using three reversals as compared with six reversals is about 0.45 dB. This increase is small compared with the overall difference in scores across conditions (with and without headset), and therefore we think that the use of three or five reversals instead of six for some of the children should not significantly affect the pattern of results.

Another possible caveat is that the order of testing of each of the four conditions was fixed across participants. This was done to make the task more predictable to the children. One could argue that, because the order of the tests was not randomized, the differences across conditions without headset and with headset could arise from learning effects. While it is not possible to rule out learning effects with the present design, differences across conditions for a closed-set task with a small number of familiar items are likely to be small. In addition, the differences found here are probably too large to be due to learning, taking into account the repeatability values reported by Summerfield et al. (1994) and Lovett et al. (2013).

One limitation of working with BC devices is the difficulties to measure their outputs. Although recently skull simulators have been incorporated to hearing-aid measurement equipment, there are still limitations: (a) The connection between the skull simulator and the device whose output is to be measured is done via a clip-on connector, similar to those used in percutaneous devices. Therefore, it is not possible to reliably measure the force of an Aftershokz headset. We have tried to do such measurements but could not obtain reliable results; (b) Even when the measurements may be carried out for some devices, audibility for individual users is difficult to estimate. Recently, Hodgetts and Scollie (2017) have described a method to fit and verify percutaneous BC devices in a skull simulator, termed the Desired Sensation Level for Bone-Conduction Devices (DSL-BCD) method. This method consists of measuring the user’s thresholds via BC through the abutment and the subsequent conversion of the dial or software levels at threshold to dB force level. After applying appropriate transforms, these levels are later used when verifying the fitting on a skull simulator. The outcomes are visualized in a graph similar to the SPLogram used for AC fittings under the Desired Sensation Level method to prescribe gain (Scollie et al., 2005). This graph is called FLogram and it is a plot of the user’s thresholds, the targets, upper limits, and output levels resulting from the fitting. Further work was done by Hodgetts, Scott, Maas, and Westover (2018) to validate a method that uses a surface microphone to verify BC fittings for BC devices including those that have skin in the vibration pathway or that have the vibrator under the skin. While initial validation was carried out for percutaneous devices, no validation was yet conducted using devices worn on a headband or fully implantable devices. These methods are still under development and are not yet validated for use in children.

It is likely that the use of a remote microphone contributed greatly to the improvement in word-discrimination threshold reported here. Direct comparisons of the BC headset paired to a microphone as used here with other technologies such as sound-field systems or remote microphones used with individual receptors that deliver the signal via AC were out of the scope of this study but could be the subject of future research. However, AC stimulation has limitations in children with OME due to AC fluctuating HL, as described earlier.

Finally, another limitation of the headset used here in that it does not have a microphone near the ears of children as BC hearing aids do. This means that the headset would not be helpful for children to communicate with their peers during the class, for example. However, if a near-the-ear microphone was included in the headset, children’s exposure to the background noise would increase, reducing the effective signal-to-noise ratio. This could be overcome by having independent volume controls (operated by the parent/teacher) for the inputs to the near-the-ear and the remote microphones.

## Summary and Conclusions

In summary, this study showed that wearing a BC headset paired with a Bluetooth microphone improved the discrimination of distant speech in quiet and in noise for children aged 3 to 6 years with OME.

## Note

1 The maximum levels of speech and noise produced by the equipment differ slightly as speech and noise were generated using different devices. Recall that while the speech was generated with the Phoenix, the noise was generated using the audiometer, as described in the text.
